# Alternative Medicine on the Internet

**DOI:** 10.2196/jmir.2.suppl2.e6

**Published:** 2000-09-13

**Authors:** Marc Muret

**Affiliations:** ^1^Zurich Physicians for Classical HomeopathySwitzerland

## Abstract

If you go to a bookstore to look for information on a particular health problem you will have a choice between the "medicine" corner with scientific manuals for professionals and the "health" corner with all kinds of books about acupuncture, ayurveda, natural healing, homeopathy, nutrition, massage, and so on! How is it on the Net? Even a short tour will bring you a lot of "medical" information, but when you look for alternative approaches in the "health" corner you will be rather disappointed. Interesting sites are rare and the amount of information very sparse. In many cases the lists of therapists are seriously incomplete; professional therapists with long experience do not appear in them. Recommendations for alternative treatments are superficial and encourage the user to buy some specialties or some book. Many sites are inflated by just quoting other sites so that, in the end, the basic information is rather poor.

As we know, "health" information is becoming increasingly important since patients want to take more responsibility for themselves. They look for alternative methods. Doctors too, as 46% of Swiss doctors use alternative methods in one way or another (Médecine et Hygiène, 1996). That is why we should not leave this part of the Internet in the hands of unqualified people. To some doctors, alternative medicine may seem a chaotic maelstrom of superstition and odd techniques. That is not so. Nearly every alternative therapy has a long tradition with its own rules and principles. All reliable therapists have undergone years of training and expect the same from their colleagues. Why should this search for quality not be present online?

What is needed? Good quality information. The identity of the author must be clear (education, tradition, professional experience, training). As many schools claim to be "the only one", the user should be informed about the differences and conflicts between all the approaches. Ethical behavior must be encouraged: respect the right to be different, show respect and tolerance, act correctly regarding copyright. More good authors should put their articles on the Net to increase the amount of basic data available online (theory, case reports, FAQ etc.). The need to create a specific (virtual) team in order to establish quality criteria, screen, control and grade the online information about alternative medicine seems obvious. This team must include professional experts with specific knowledge and experience. A homeopath should evaluate homeopathy, an acupuncturist should evaluate acupuncture. Serious thought has to be given to creating a central data bank on alternative medicine, to provide quality information to different portals and websites, as well as to patients, doctors, educators and journalists.

